# LINC02878/ZNF282/PYCR2 axis promotes proline synthesis and tumor progression in colorectal cancer

**DOI:** 10.1007/s00018-025-05968-3

**Published:** 2025-12-02

**Authors:** Jun-ping Lei, Lin Wang, Tian-yang Wang, Bing-hu Lin, Yan Xu, Song Shang, Tian Xia, Min-ming Gong, Yu Guo, Jia Fu, Yuan-lin Li

**Affiliations:** 1 Department of Colorectal and Anal Surgery, Hubei University of Medicine, Xiangyang No.1 People’s Hospital, Xiangyang, 441000 China; 2 Department of Gastroenterology, People’s Hospital, Hubei University of Medicine, Xiangyang No.1, Xiangyang, 441000 China; 3 Department of Pulmonary and Critical Care Medicine, People’s Hospital, Hubei University of Medicine, Xiangyang No.1, Xiangyang, 441000 China; 4 Department of Medical Insurance Price Management Office, People’s Hospital, Hubei University of Medicine, Xiangyang No.1, Xiangyang, 441000 China

**Keywords:** LINC02878, ZNF282, PYCR2, Proline synthesis, Colorectal cancer

## Abstract

Colorectal cancer (CRC) remains a leading cause of cancer-related mortality globally, with poor survival outcomes in advanced stages due to therapy resistance and metastasis. While long non-coding RNAs (lncRNAs) are emerging as key regulators of CRC progression, their functional interplay and metabolic implications remain poorly understood. Here, we identified LINC02878 as a novel oncogenic lncRNA significantly upregulated in CRC and correlated with poor prognosis. Mechanistically, LINC02878 binds to the ZNF282 to activate PYCR2 expression, thereby enhancing proline biosynthesis and driving tumor aggressiveness. In vivo, LINC02878 knockdown suppressed subcutaneous tumor growth, reduced lung metastasis, and improved survival in xenograft models. Our study unveils the LINC02878/ZNF282/PYCR2/proline axis as a critical metabolic vulnerability in CRC, offering potential therapeutic targets for intervention.

**Supplementary Information:**

The online version contains supplementary material available at 10.1007/s00018-025-05968-3.

## Introduction

Colorectal cancer (CRC) is the third leading cause of morbidity and the second leading cause of mortality worldwide [1]. According to the Global Cancer Statistics 2022, there were 1,926,118 new cases diagnosed worldwide in 2022, resulting in 903,859 deaths [1]. Currently, comprehensive treatments, including surgery, chemotherapy, and targeted therapy, are available in clinical practice [2]. However, CRC remains the most lethal disease, primarily due to advanced stages, chemotherapy resistance, and recurrence and metastasis [2]. Research indicated that among patients with advanced or metastatic CRC, fewer than one-fifth survive beyond five years [3]. Thus, investigating the molecular mechanisms underlying the malignant progression of CRC can contribute to facilitating the identification of new therapeutic targets and improve patient survival outcomes.

Long non-coding RNAs (lncRNAs) are a class of non-coding RNA that exceed 200 nucleotides in length, which can activate or inhibit the expression of specific genes through various mechanisms, including transcription and translation, thereby influencing cellular functions [4]. Researches have indicated that various lncRNAs function as carcinogenic regulators in CRC [5–7]. For instance, small nucleolar RNA host gene 4 (SNHG4) is a lncRNA that has been reported to act as an oncogene in CRC [5]. Ring Finger Protein 14 (RNF14) is an E3 ubiquitin ligase involved in protein degradation and signaling regulation in CRC, RNF14 overexpression is associated with poor prognosis and contributes to tumor growth by modulating key oncogenic pathways, such as Wnt/β-catenin signaling [5]. Recently research reported that SNHG4 promotes tumor progression through sustained activation of oncogenic pathways such as Wnt/β-catenin signaling by stabilizing RNF14 mRNA through interaction with the RNA-binding protein TBP-associated factor 15 (TAF15), thereby enhancing cell proliferation and metastasis [5]. RNF14 mRNA by recruiting the RNA-binding protein TAF15, thereby promoting CRC progression through sustained activation of oncogenic pathways such as Wnt/β-catenin signaling. LOXL1 antisense RNA 1 (LOXL1-AS1) enhances the cancer progression of CRC by regulating the miR-1224-5p/miR-761/hexokinase 2 (HK2) axis [6]. lncRNA targeting antisense sequence of SP100 gene (SP100-AS1) facilitates CRC radiotherapy resistance by sponge miR-622 and stabilizing ATG3 [7]. More importantly, lncRNAs are also important tumor markers for CRC [4]. Although the human genome is estimated to encode over 28,000 different lncRNAs, the vast majority of which remain uncharacterized and unannotated according to the ENCODE project [8]. Thus, investigating novel lncRNAs may identify key biomarkers and potential therapeutic targets for CRC, playing a crucial role in early diagnosis, targeted therapy, and prognosis assessment.

Transcription factors (TFs) are a class of proteins that play a regulatory role in transcription within the nucleus and are crucial for the regulation of cell growth, proliferation, differentiation, and invasion metastasis [9]. Studies have found that multiple TFs play oncogenic roles in CRC [10–12]. For example, SRY-box transcription factor 2 (SOX2) promotes chemoresistance, cancer stem cell properties, and epithelial-mesenchymal transition in CRC [10]. KLF transcription factor 7 (KLF7) enhances the progression of CRC cells through the miR-139-5p/tumor protein D52 (TPD52) axis [11]. Zic family zinc finger 2 (ZIC2) stimulates the growth and metastasis of CRC through the TGF-β signaling pathway [12]. Zinc finger protein 282 (ZNF282) is a recently identified TF that is thought to play an oncogenic role in both breast cancer and esophageal cancer [13, 14]. Mechanistically, ZNF282 functions as a critical coactivator of E2F transcription factor 1(E2F1), transcriptionally upregulating the expression of cyclin A1 (CCNA1) and cell division cycle 6 (CDC6), thereby driving malignant progression in esophageal squamous cell carcinoma (ESCC) [13]. SUMOylation of ZNF282 augments its coactivator function through enhanced binding interactions with ERα and the coiled-coil coactivator (CoCoA), leading to transcriptional upregulation of ERα target genes (TFF1/pS2, GREB1, PgR, and CTSD) and consequent stimulation of estrogen-dependent breast cancer cell proliferation [14]. However, the role of ZNF282 in CRC remains unclear.

Proline is the only proteogenic secondary amino acid abundant in the body [15]. As a non-essential amino acid, it plays a role in various biological pathways relevant to cancer, including protein biosynthesis, redox homeostasis, and immune evasion [15]. For example, the MZF1 antisense RNA 1(MZF1-AS1)/poly (ADP-ribose) polymerase 1 (PARP1)/E2F1 axis enhances the malignant progression of neuroblastoma by promoting the synthesis of proline [16]. Proline synthesized by proline synthetase pyrroline-5-carboxylate reductase 1 (PYCR1) activates the cGMP-PKG signaling pathway, which is critical for breast cancer stemness and tumor growth [17]. Furthermore, sirtuin 3 (SIRT3) regulates cancer cell proliferation and proline metabolism by deacetylating the K228 site of the PYCR1 [18]. Although research have demonstrated that increased proline biosynthesis is closely associated with the malignant progression of CRC [19, 20], the underlying molecular mechanisms remain unclear.

In this study, we demonstrated that LINC02878 is significantly upregulated in CRC and associated with poor prognosis. Mechanistically, LINC02878 exerts its oncogenic effects by binding to the ZNF282 and upregulating pyrroline-5-carboxylate reductase 2 (PYCR2) expression, thereby enhancing proline biosynthesis. Notably, in vivo experiments revealed that LINC02878 knockdown suppressed subcutaneous tumor growth, reduced pulmonary metastatic nodule formation in tail vein-injected nude mice, and prolonged survival. Collectively, our results uncover a novel role of LINC02878 in reprogramming proline metabolism in CRC and highlight its potential as a therapeutic target for CRC treatment.

## Materials and methods

###  Bioinformatics analysis

Gene expression profiles and corresponding clinical data of colorectal adenocarcinoma (COADREAD) were obtained from The Cancer Genome Atlas (TCGA) portal (https://portal.gdc.cancer.gov/) (including 647 tumor samples and 51 normal tissue samples). Raw expression data were log2-transformed and normalized using the “impute” package for missing value estimation and the “limma” package for quantile normalization and variance stabilization. Furthermore, univariate and multivariate regression analyses were conducted using the “survival” and “rms” packages. and genetic difference analysis was performed using the “limma” package. Gene Ontology (GO), Kyoto Encyclopedia of Genes and Genomes (KEGG) and Gene Set Enrichment Analysis (GSEA) enrichment analyses were performed using the “clusterProfiler” and “msigdbr” packages. Additionally, 67 genes related to arginine and proline metabolism were obtained from GSEA (https://www.gsea-msigdb.org/) (GOBP_PROLINE_METABOLIC_PROCESS, KEGG_ARGININE_AND_PROLINE_METABOLISM). The JASPAR dataset [21] was used for the prediction of TF binding sites.

### Cell culture

A normal human colon epithelial cell line (CP-H040) and five CRC (CRC) cell lines (HCT15, HCT116, LoVo, LS513, and SW480) were obtained from Procell Life Science & Technology Co., Ltd (Wuhan, China). All cell lines were maintained in either RPMI 1640 or DMEM medium, supplemented with 10% fetal bovine serum, at 37 °C in a humidified 5% CO_2_ incubator.

### CCK-8 assay

Cell viability was assessed using the Cell Counting Kit-8 (G4103, Servicebio, Wuhan) according to the manufacturer’s protocol [22]. Briefly, cells were seeded in 96-well plates at a density of 3 × 10^3^ cells/well and allowed to adhere overnight. 10 µL of CCK-8 reagent was added to each well followed by incubation at 37 °C for 2 h. Absorbance was measured at 450 nm using a microplate reader. Cell viability was calculated as: (OD_treatment_-OD_blank_)/(OD_control_-OD_blank_)×100%. All experiments were performed in triplicate wells and repeated three times independently.

### Edu assay

Cell proliferation was evaluated using the Edu-555 Kit (G1602, Servicebio, Wuhan) following the manufacturer’s protocol with modifications and previous literature reports [23]. Briefly, cells were seeded in 24-well plates (2 × 10^4^ cells/well) and cultured for 24 h. Actively proliferating cells were labeled with 10 µM EdU (5-ethynyl-2’-deoxyuridine) for 2 h at 37 °C. Cells were fixed with 4% paraformaldehyde (15 min) and permeabilized 20 min with 0.5% Triton X-100 (versus the recommended 15 min) to enhance probe penetration while preserving cellular morphology. EdU incorporation was detected by incubating with Edu-555 (30 min, dark), followed by nuclear counterstaining with Hoechst 33,342 to 10 min (versus the standard 5 min) to ensure uniform chromatin labeling intensity across all samples. Images were acquired using a fluorescence microscope. Five random fields per well were analyzed with ImageJ to calculate the EdU-positive rate (% labeled nuclei/total nuclei). Three biological replicates were performed.

### Cell migration and invasion assays

Cell migratory capacity was evaluated using Transwell inserts (Corning, #3422, 8-µm). Briefly, serum-starved cells (2 × 10^4^ cells in 200 µL serum-free medium) were seeded into the upper chamber. The lower chamber was filled with 600 µL complete medium containing 10% FBS as chemoattractant. After 24 h incubation (37 °C, 5% CO₂), non-migrated cells on the upper membrane surface were removed with cotton swabs. Migrated cells were fixed with 4% paraformaldehyde (15 min), stained with 0.1% crystal violet (20 min), and quantified by counting five random fields per insert under microscopy. For invasion assessment, Transwell inserts were pre-coated with Matrigel (Corning, #356234, 1:8 dilution in serum-free medium, 100 µL/insert, polymerized for 4 hr at 37 °C). The remaining protocol matched the migration assay. Images were processed using ImageJ with the Cell Counter plugin. Data were normalized to control groups and expressed as mean ± SD of migrated/invaded cells per field. Three independent experiments were performed in technical triplicate.

### Proline and NAD/NADH level

Intracellular proline levels and NAD/NADH ratio were quantified using commercial assay kits according to the manufacturers’ protocols. Specifically, proline concentration was determined using the Proline Content Assay Kit (BC0290, Solarbio, Beijing) [24], while NAD/NADH ratio were measured using a NAD/NADH Assay Kit (S0175, Beyotime, Shanghai) according to the manufacturers’ protocols [25].

### Reverse transcription-quantitative polymerase chain reaction (RT-qPCR)

The primers used in this study were designed using Oligo software (version 7.37) [26] and validated for specificity through NCBI BLAST analysis. Primer performance was further confirmed by generating standard curves with five-point, 10-fold serial dilutions of cDNA. PCR efficiency (E) was calculated according to the equation:

E=(10^(−1/*slope)*^−1)×100%.

All primers exhibited amplification efficiencies between 90% and 110% with correlation coefficients (R²) > 0.99. The sequences, product lengths, sources, and corresponding PCR efficiencies are summarized in Table S1.

In short, total RNA was extracted using TRIzol reagent (Invitrogen) and reverse-transcribed into cDNA with the PrimeScript RT reagent kit (RR037A, Takara). qPCR was performed using SYBR Green Master Mix (11201ES, Yeasen, Shanghai) on a QuantStudio 5 system (Applied Biosystems) [27]. Reactions (20 µL) contained 1 µL cDNA, 0.5 µM primers, and 10 µL Master Mix. Cycling conditions: 95 °C for 30 s, followed by 40 cycles of 95 °C for 5 s and 60 °C for 30 s. Melt curve analysis verified specificity. Relative expression was calculated by the 2^−ΔΔCt^ method using β-actin as the endogenous control.

### Western blot

In summary, cells were lysed in RIPA buffer containing protease inhibitors, and the supernatant was collected. Protein concentration was measured using the BCA assay, and samples were normalized accordingly. Protein samples were then mixed with loading buffer and heated at 100 °C for 10 min. Equal amounts (50 µg) were loaded onto a 10% SDS-PAGE gel, and the proteins were transferred to a PVDF membrane. Blocking was performed at room temperature for 1 h using 5% non-fat milk in TBST. The primary antibodyanti-PYCR2 (1:5000, 17146-1-AP, Proteintech), anti-ZNF282 (1:1000, HPA024374, Merck), and anti-β-actin (1:2000, GB15003, Servicebio) was incubated overnight at 4 °C, followed by a 1-hour incubation with the secondary antibody. The chemiluminescent signals were captured using an imaging system.

### Gene overexpression and knockdown

The overexpression vectors for human LINC02878 (Gene ID: 286076), ZNF282 (Gene ID: 8427), and PYCR2 (Gene ID: 29920), alongside an empty vector, and the shRNAs targeting LINC02878, ZNF282 and PYCR2, were purchased from Wzbio Technology (Jinan, Shandong. The target shRNA sequences were as follows: LINC02878 shRNA#1: ACGCTCTTGTGCAGGATTAAA; LINC02878 shRNA#2: GGTCGTTAGTCCCTCACAATG; ZNF282 shRNA#1: GACCTCTTGTCCCGGATTAAA; ZNF282#2: GTTCCCACCCTTTCCAGATAG; PYCR2 shRNA#1: CTGTCGGCTCACAAGATAATA; and PYCR2 shRNA#2: GCAGCATCCATGCCAGCTTAA.

For lentiviral transduction, cells were plated in 6-well plates (3 × 10⁵ cells/well) and infected at 30–50% confluence using Hitrans G reagent. For co-transfection, overexpression vectors carried puromycin resistance, while knockdown vectors contained G418 resistance. Stable cell lines were established by selection with 2 µg/mL puromycin or 400 µg/mL G418 for 7–10 days, and gene modification was verified by RT-qPCR and Western blot [27].

### RNA sequencing analysis

Total RNA was extracted from stable transfected cancer cells (1 × 10^6^) using TRIzol™ reagent (Life Technologies, Gaithersburg, MD). RNA-seq libraries were prepared following standard Illumina protocols and sequenced on an Illumina HiSeq X Ten platform (Novogene Bioinformatics Technology Co., Ltd., Beijing, China) to generate 100-bp paired-end reads. Read alignment and quantification were performed using HTSeq v0.6.0 to determine the number of reads mapped to each gene. Gene expression levels were normalized and expressed as fragments per kilobase of transcript per million mapped reads (FPKM).

### Dual-luciferase reporter, chip and qPCR assays

The promoter region of human PYCR2 (Gene ID: 29920, spanning nucleotides − 2000 to + 99 relative to the transcription start site) was amplified and cloned into the pGL6-dura luciferase reporter vector (D2091, Beyotime) to quantitatively assess promoter activity through dual-luciferase reporter assays. For analysis of the PYCR2 promoter (−1220/−1076), the following high-specificity primers were designed and validated: Forward: 5’-GGTGTGCATTACACGGAAAGGA-3, ‘Reverse: 5’-AGTAAATATAAGGGGCCGCAGG-3’. In addition, chromatin immunoprecipitation was performed using the commercial ChIP assay kit (P2078, Beyotime) following the manufacturer’s optimized protocol [28]. The enriched PYCR2 promoter fragment (−577/−426) was quantified using qPCR with the following primers: Forward: 5’-GCCACCTGGTCTGAACCTCG-3’. Reverse: 5’-GGCCCAGGCCTGGCTAATAT-3’. Luciferase assays included triplicate technical replicates and normalization to Renilla luciferase activity. ChIP-qPCR results were calculated as fold enrichment relative to IgG control and normalized to input DNA.

### 12 RNA fluorescence in situ hybridization (RNA-FISH) and Immunofluorescence assays

RNA-FISH was performed using target-specific Cy3-labeled probes (BersinBio, Guangzhou) according to the manufacturer’s protocol. Briefly, cells were fixed in 4% paraformaldehyde (PFA), permeabilized with 0.5% Triton X-100, and hybridized with probes overnight at 37 °C. Nuclei were counterstained with DAPI (1 µg/mL). For immunofluorescence, fixed cells were blocked with 5% BSA and incubated with primary antibodies (1:200 dilution) at 4 °C overnight, followed by FITC-conjugated secondary antibodies (1:500) for 1 h at room temperature. Images were acquired using a confocal microscope.

### RNA pull-down and RNA Immunoprecipitation (RIP)

RNA pull-down and RIP assays were conducted as described previously [29]. Biotin-labeled RNA probes were synthesized in vitro and incubated with cell lysates at 4 °C for 2 h. Streptavidin magnetic beads were then added to capture RNA-protein complexes. After extensive washing, bound proteins were eluted and analyzed by Western blot. For RIP assay, cells were lysed in RIP buffer and incubated with antibody-coated magnetic beads overnight at 4 °C. IgG was used as negative control. RNA-protein complexes were immunoprecipitated, followed by RNA extraction. Precipitated RNAs were quantified by RT-qPCR.

### Hematoxylin & Eosin (H&E) and immunohistochemistry (IHC) staining

Tissue Sect. (4 μm) were deparaffinized, rehydrated, and stained with hematoxylin for 5 min followed by eosin for 1 min. After dehydration, slides were mounted with neutral balsam and imaged under bright-field microscopy. For IHC Staining, antigen retrieval was performed in citrate buffer (pH 6.0) at 95 °C for 15 min. Endogenous peroxidase was blocked with 3% H_2_O_2_ for 10 min. Sections were incubated with anti-ZNF282 (1:200, PH6638, Abmart), anti-PYCR2 (1:100, A15155, Abclonal), anti-Ki-67 (1:200, GB111499, Servicebio) overnight at 4 °C, followed by HRP-conjugated secondary antibodies (1:500, GB23303, Servicebio) for 1 h at room temperature. DAB substrate was used for color development, and hematoxylin counterstaining was applied. In this study, quantitative analysis of IHC was performed using average optical density (AOD) as the primary metric. AOD values were calculated by dividing integrated optical density (IOD) by the corresponding area of interest (AOD = IOD/area) [30].

### Animal experiment

100µL cancer cells (5 × 10^7^/ml) stably transfected were subcutaneously injected into the flanks of 6-week-old BALB/c nude mice (*n* = 5/group).Tumor dimensions were measured weekly using calipers, and volume was calculated as (length×width^2^)/2. Mice were sacrificed at 5 weeks post-injection for tumor excision and weighing.

For experimental lung metastasis model, 100µL tumor cells (1 × 10^7^/ml) resuspended with PBS were intravenously injected via the tail vein of nude mice (*n* = 5/group). After 5 weeks, mice were euthanized and lungs were harvested. Metastatic nodules were quantified by hematoxylin & eosin (H&E) staining of paraffin-embedded sections. All procedures were approved by the Ethics Committee of Xiangyang No.1 People’s Hospital.

### Human tissues

This human research was approved by the Ethics Committee of Xiangyang No.1 People’s Hospital. All patients provided written informed consent prior to surgery and had not undergone any treatment in the three months preceding tissue collection. Histopathological examination confirmed the diagnosis of CRC. We collected 50 paired CRC and adjacent normal colorectal tissue samples to quantify mRNA expression levels of LINC02878, ZNF282, and PYCR2 using RT-qPCR. Protein levels of ZNF282 and PYCR2 were analyzed in 20 paired CRC and normal tissue samples by Western blotting. ZNF282 and PYCR2 protein expression was assessed in 92 CRC cases and matched normal tissues using IHC. Staining intensity was quantified using ImageJ software [30].

### Statistical analysis

All experiments were independently replicated a minimum of three times to ensure reproducibility. Prior to statistical comparisons, we evaluated data distribution characteristics (normality and homogeneity of variance) using GraphPad Prism software. Based on these assessments, appropriate parametric or non-parametric tests were selected: Normally distributed data with two-tailed unpaired Student’s t-test, non-normally distributed data with Mann-Whitney U test, respectively. One-way ANOVA with Bonferroni’s post hoc correction for multiple group comparisons. Survival outcomes were analyzed using log-rank testing. Correlation analyses were performed using Pearson’s method for normally distributed continuous variables. All statistical analyses were conducted using GraphPad Prism 10.0, with a predetermined significance threshold of *P* < 0.05.

## Results

### LINC02878 is significantly upregulated in CRC and associated with poor prognosis

**Fig. 1:**
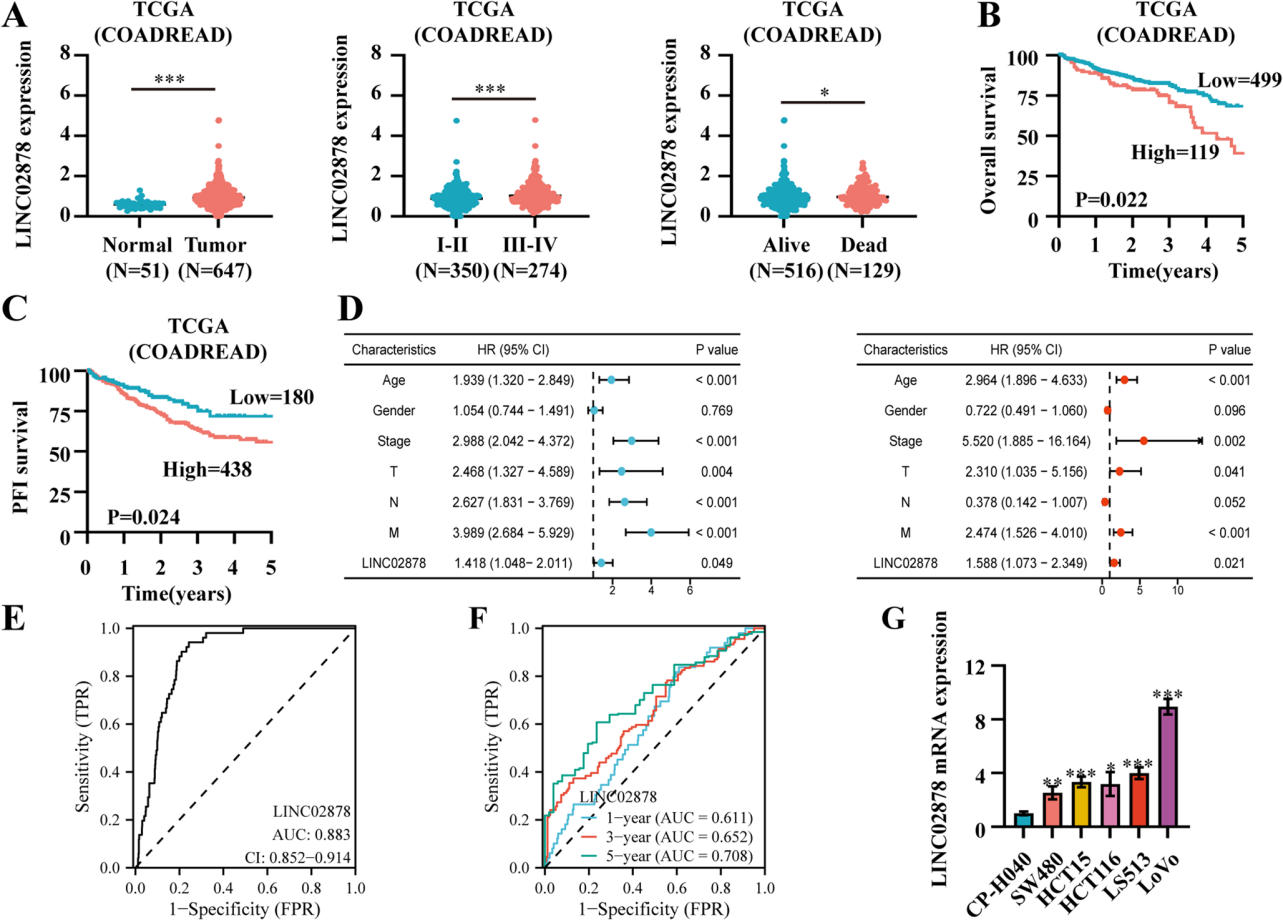
LINC02878 is significantly upregulated in CRC and associated with poor prognosis.** (A)** The expression of LINC02878 is significantly increased in tumor tissues (left panel), Ⅲ-Ⅳ stage (middle panel) and death groups (right panel) based on the TCGA-COADREAD. (**B-C**) The K-M curve suggesting the overall survival (OS) and progression free survival (PFI) of LINC02878 based on the TCGA-COADREAD, respectively. (**D**) Forest plots showing univariate and multivariate regression analyses. (**E-F**) ROC curves demonstrating the diagnostic (**E**) and prognostic (**F**) efficacy of LINC02878, respectively. (**G**) RT-qPCR detecting the mRNA expression levels of LINC02878 in five types of CRC cells (HCT15, HCT116, LoVo, LS513, and SW480) and normal colonic mucosal epithelial cells (CP-H040) (*n* = 3). *P < 0.05, **P < 0.01, ***P < 0.001

First, we investigated the clinical relevance of LINC02878 in CRC based on the TCGA-COADREAD cohort. Our analysis revealed significantly elevated LINC02878 expression levels in CRC tissues compared to normal tissues (Fig. 1A). Notably, higher expression was associated with Ⅲ-Ⅳ stages and correlated with patient mortality (Fig. 1A). Furthermore, elevated LINC02878 expression demonstrated significant prognostic value, showing strong associations with both poorer overall survival (OS) and progression-free interval (PFI) in CRC patients (Fig. 1B-C). Moreover, to assess the prognostic significance of LINC02878 in CRC, we performed univariate and multivariate Cox regression analyses based on the TCGA-COADREAD cohort. The results revealed that LINC02878 serves as an independent prognostic factor for CRC, even after adjusting for potential confounding variables (Fig. 1D). Notably, receiver operating characteristic (ROC) curve analysis suggested that LINC02878 exhibits significant diagnostic utility and prognostic predictive value in CRC, highlighting its potential clinical relevance (Fig. 1E-F). To validate LINC02878 expression patterns in CRC, we performed RT-qPCR analysis across five CRC cell lines (HCT116, HCT15, LoVo, LS513, and SW480) and normal human colonic mucosal epithelial cells (CP-H040) (Fig. 1G). Our results revealed consistently elevated LINC02878 expression in all malignant cell lines compared to normal cell line (CP-H040), with LoVo cells demonstrating the highest expression levels. In summary, our results indicated that LINC02878 is consistently upregulated in CRC and strongly correlates with adverse clinical outcomes.

### LINC02878 promotes the cell viability, proliferation, migration and invasion ability of CRC cells in vitro

**Fig. 2:**
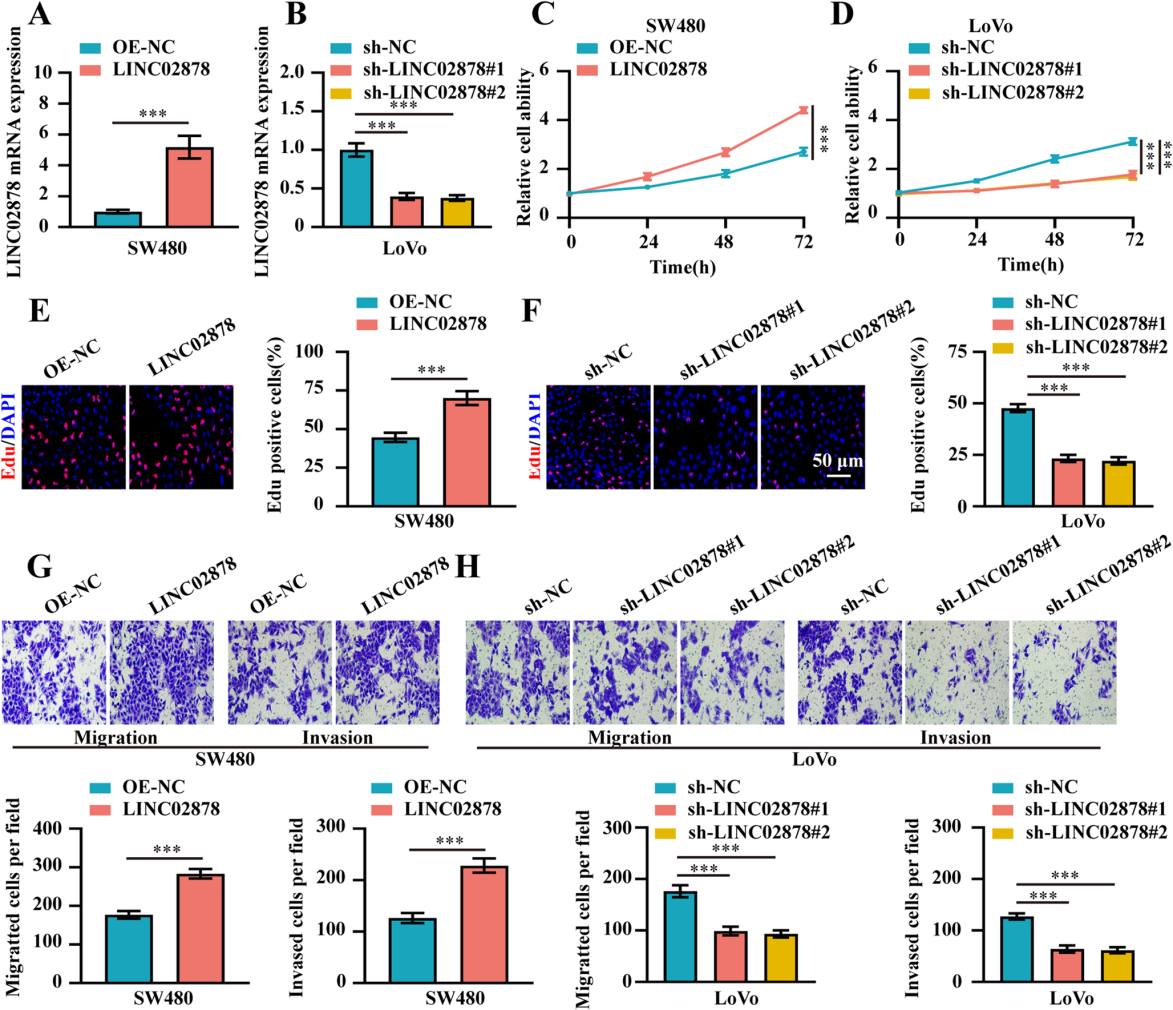
Overexpression of LINC02878 promotes the cell viability, proliferation, migration and invasion ability of CRC cells. **(A-B)** RT-qPCR evaluating the mRNA of LINC02878 in SW480 and LoVo cells transfected as indicated, respectively (*n* = 3). (**C-D**) CCK-8 detecting the cell viability of SW480 and LoVo cells transfected as indicated, respectively (*n* = 3). (**E-F**) EdU assay evaluating the cell proliferation ability of SW480 and LoVo cells transfected as indicated, respectively (*n* = 3). (**G-H**) Transwell migration and invasion assay detecting the cell migration and invasion ability of SW480 and LoVo cells transfected as indicated, respectively (*n* = 3). *P < 0.05, **P < 0.01, ***P < 0.001

Based on the differential expression profile of LINC02878 across CRC cell lines, we selected SW480 (lower expressor) and LoVo (highest expressor) as representative models for subsequent functional investigations (Fig. 1G). First, we established stable LINC02878-knockdown models in LoVo cell lines and stable LINC02878-overexpression models in SW480 cell lines, using lentiviral transfection and transduction efficiency was confirmed by RT-qPCR (Fig. 2A-B). Functional assays demonstrated that LINC02878 overexpression in SW480 cells significantly promoted oncogenic phenotypes, including enhanced cell viability, proliferation, migration, and invasion ability (Fig. 2C, E, **and G**). Conversely, LINC02878 knockdown in LoVo cells markedly suppressed these malignant behaviors (Fig. 2D, F, **and H**).

### LINC02878 regulates proline metabolism in CRC through PYCR2

**Fig. 3:**
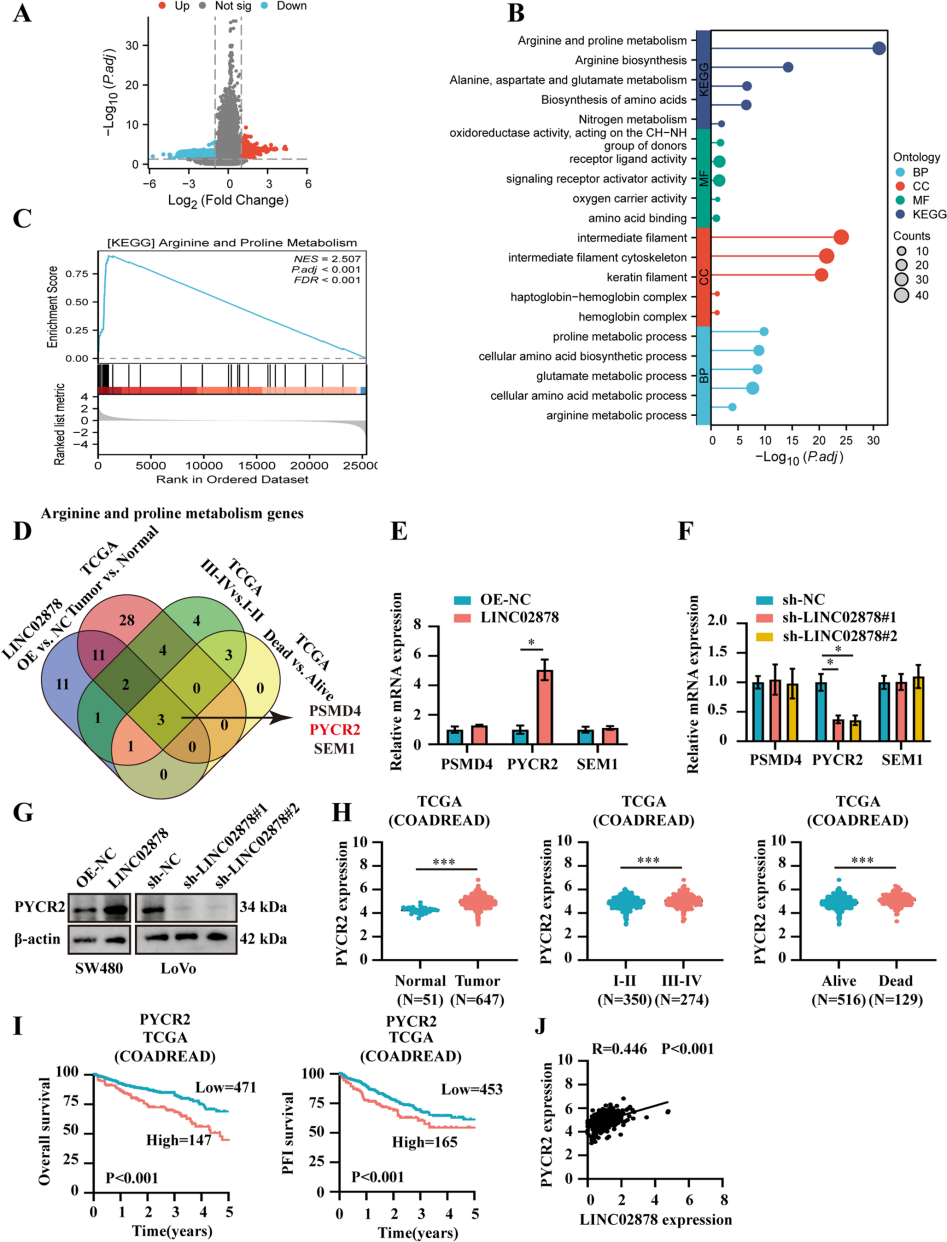
LINC02878 regulates proline metabolism in CRC through PYCR2.** (A)** Volcano plot illustrating the differential gene expression profile in SW480 cells following stably overexpression of LINC02878 (fold change > 2, FDR < 0.05). (**B**) Lollipop chart showing the GO and KEGG enrichment analysis results of differentially expressed genes. (**C**) GSEA identifying significant pathway enrichment upon stable LINC02878 overexpression. (**D**) Venn diagrams indicating the differences genes of arginine and proline metabolism based on RNA-seq and TCGA-COADREAD as indicated. (**E-F**) RT-qPCR evaluating the mRNA of target genes in SW480 and LoVo cells transfected as indicated, respectively (*n* = 3). (**G**) Western blot evaluating the protein of PYCR2 in SW480 and LoVo cells transfected as indicated, respectively (*n* = 3). (**H**) The expression of PYCR2 is significantly increased in tumor tissues (left panel), Ⅲ-Ⅳ stage (middle panel) and death groups (right panel) based on the TCGA-COADREAD. (**I**) The K-M curve suggesting the overall survival (OS) and progression free survival (PFI) of PYCR2 based on the TCGA-COADREAD, respectively. (**J**) Scatter plot showing the correlation of the expression levels of LINC02878 and PYCR2 based on the TCGA-COADREAD, respectively. *P < 0.05, **P < 0.01, ***P < 0.001

To elucidate the oncogenic mechanisms mediated by LINC02878 in CRC, we performed transcriptome profiling via RNA-seq on SW480 cells stably overexpressing LINC02878. Comparative analysis revealed 604 significantly upregulated and 506 downregulated genes (|fold change| >2, FDR < 0.05) in LINC02878-overexpressing cells relative to empty vector controls (OE-NC group) (Fig. 3A). Functional enrichment analysis of the differentially expressed genes demonstrated significant involvement in arginine and proline metabolism pathways, as evidenced by both GO and KEGG analyses (Fig. 3B). This finding was further corroborated by GSEA, which showed consistent enrichment patterns (Fig. 3C). Subsequently, we performed an integrated differential expression analysis incorporating data from TCGA-COADREAD. Our analysis identified that 29 differentially expressed genes (DEGs) following LINC02878 overexpression in LoVo cells, and 48 DEGs in tumor versus normal tissues,18 stage-associated DEGs, and 7 survival-related DEGs in deceased patients based on the TCGA-COADREAD, respectively (Fig. 3D). Intersection analysis revealed three consistently dysregulated genes (PSMD4, PYCR2, and SEM1) across all comparison sets (LINC02878-overexpressing LoVo cells, TCGA tumor samples, stage groups, and survival cohorts), suggesting their potential as core downstream effectors of LINC02878-mediated oncogenesis (Fig. 3D, Figure S1). To systematically identify LINC02878 target genes, we performed RT-qPCR validation of candidate genes following LINC02878 modulation. Our results analysis demonstrated that PYCR2 expression exhibited significant positive correlation with LINC02878 levels, whereas PSMD4 and SEM1 expression remained unchanged, suggesting PYCR2 as a specific downstream target of LINC02878-mediated regulation (Fig. 3E-F). Western Blot also confirmed the PYCR2 expression pattern regulating by LINC02878 (Fig. 3G). Moreover, analysis of TCGA-COADREAD data revealed that PYCR2 expression was also significantly elevated in tumor tissues, advanced-stage cases, and among deceased patients. Additionally, high PYCR2 expression correlated with poorer OS and PFI, consistent with the significance of LINC02878 (Fig. 3H-I). More importantly, the correlation analysis showed that the expression level of PYCR2 was significantly positively correlated with LINC02878 according to the TCGA-COADREAD (Fig. 3J, *R* = 0.446, *P* < 0.001).

### LINC02878 promotes cancer progression of CRC depend on PYCR2

**Fig. 4:**
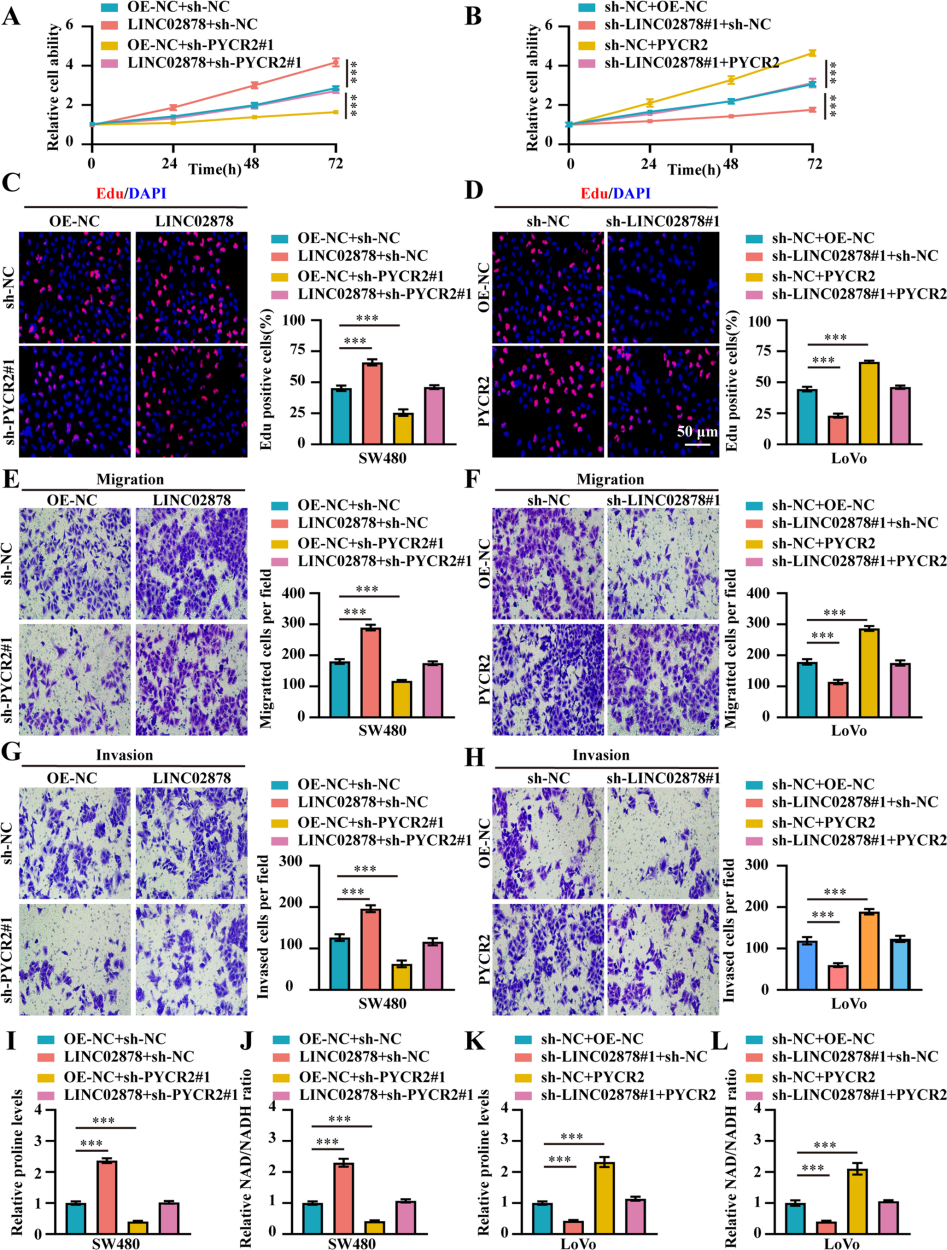
LINC02878 promotes cancer progression and proline metabolism of CRC depend on PYCR2. **(A-B)** CCK-8 detecting the cell viability of SW480 and LoVo cells transfected as indicated, respectively (*n* = 3). (**C-D**) EdU assay evaluating the cell proliferation ability of SW480 and LoVo cells transfected as indicated, respectively (*n* = 3). (**E-H**) Transwell migration and invasion assay detecting the cell migration and invasion ability of SW480 and LoVo cells transfected as indicated, respectively (*n* = 3). (**I-L**) The proline levels, and NAD/NADH ratio in SW480 and LoVo cells stably transfected as indicated, respectively (*n* = 3). *P < 0.05, **P < 0.01, ***P < 0.001

To elucidate the functional dependency of LINC02878-mediated oncogenic effects on PYCR2, we conducted a series of rescue experiments in CRC models. Functional rescue experiments demonstrated that knockdown of PYCR2 significantly attenuated the oncogenic effects mediated by LINC02878 in SW480 cells, reversing the increases in cell viability, proliferation, migration, and invasion induced by LINC02878 overexpression (Fig. 4A, C, E, **and G**). Conversely, overexpression of PYCR2 restored malignant phenotypes in LINC02878-knockdown LoVo cells, rescuing the impaired cell viability, proliferation, migration, and invasion capacity resulting from LINC02878 silencing (Fig. 4B, D, F, **and H**). Furthermore, we also investigated the effect of LINC02878 on promoting proline and NAD/NADH ratio through PYCR2. Our results revealed that LINC02878 overexpression significantly enhanced proline biosynthesis and NAD/NADH ratio in SW480 cells (Fig. 4I-J). Notably, PYCR2 knockdown completely abrogated these LINC02878-mediated metabolic effects, reducing both proline production and NAD/NADH ratio (Fig. 4I-J). On the other hand, stable knockdown of LINC02878 significantly inhibited the levels of proline and NAD/NADH ratio in LoVo cells, while stable overexpression of PYCR2 rescued the inhibitory effect of knockdown of LINC02878 on proline synthesis and NAD/NADH ratio in LoVo cells (Fig. 4K-L), establishing PYCR2 as the crucial proline metabolic effector downstream of LINC02878.

### LINC02878 regulates PYCR2 by binding to ZNF282 in CRC cells

**Fig. 5:**
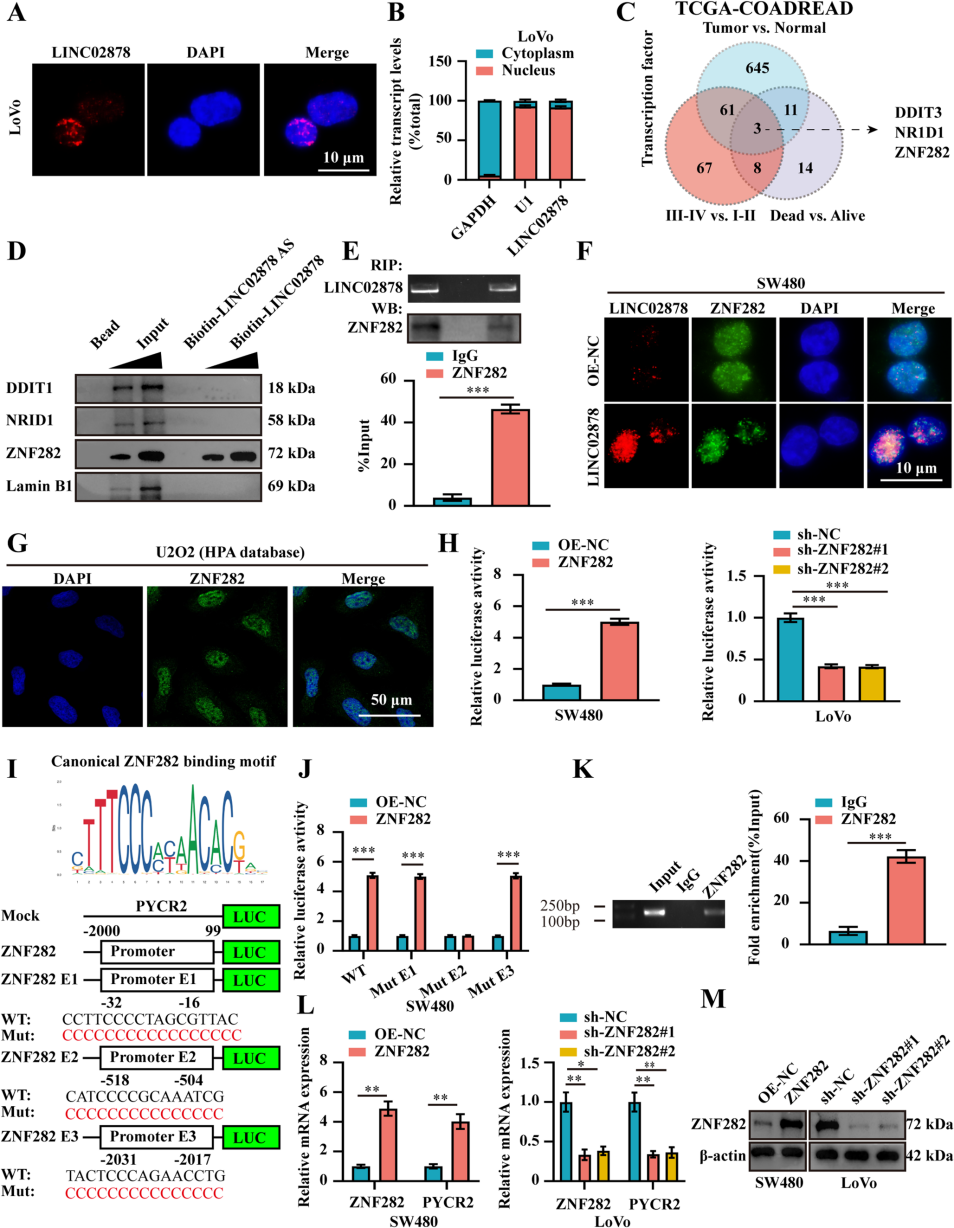
LINC02878 regulates PYCR2 by binding to ZNF282 in CRC Cells. (**A-B**) RNA-FISH (**A**) and nuclear plasma separation (**B**) assays evaluating the cell sub-localization of LINC02878 in LoVo cells, respectively (*n* = 3). (**C**) The differential TFs are analyzed based on the TCGA-COADREAD dataset. (**D**) Western blot analysis following biotinylated RNA pull-down assays confirmed specific protein interactions with LINC02878 in LoVo cells, with appropriate negative controls (bead-bound proteins and LINC02878 AS RNA). (**E**) RNA immunoprecipitation (RIP, upper panel) and Western blot assays (lower panel) indicating physical interaction of LINC02878 with ZNF282 protein in LoVo cells (*n* = 3). (**F**) Immunofluorescence confocal imaging demonstrating significant co-localization between LINC02878 (red fluorescence) and ZNF282 protein (green fluorescence) within the nuclear compartment of SW480 cells. (**G**) Immunofluorescence showing that the ZNF282 protein was mainly located in the nucleus of U2O2 cells based on the HPA database. (**H**) Luciferase reporter assays are performed to assess PYCR2 promoter transcriptional activity in SW480 and LoVo cells following transfection with the indicated constructs (*n* = 3). (**I**) Bioinformatic analysis using the JASPAR database (https://jaspar.genereg.net/) revealed the consensus binding motif of ZNF282 and predicted three potential ZNF282-responsive elements in the PYCR2 promoter region. (**J**) Luciferase reporter assays are performed to assess the transcriptional activity of the three PYCR2 promoter mutants upon ZNF282 overexpression in SW480 cells (*n* = 3). (K) ChIP assays confirmed ZNF282 binding to the PYCR2 promoter, with IgG serving as the negative control (*n* = 3). (**L-M**) RT-qPCR (**L**, *n* = 3) and western blot (**M**, *n* = 3) evaluating the mRNA and protein levels of ZNF282 and PYCR2 after stably transfected as indicated, respectively. *P < 0.05, **P < 0.01, ***P < 0.001

To further clarify the molecular mechanism by which LINC02878 regulates PYCR2, we first explored the cellular sub-localization of LINC02878 in CRC cells. We found that LINC02878 is mainly located in the nucleus based on the RNALocate v2.0 database [31]. RNA-FISH and nuclear/cytoplasmic RNA assays consistently confirmed that LINC02878 was mainly located in the nucleus of LoVo cells (Fig. 5A-B). Based on the established lncRNA mechanisms [32], we propose that LINC02878 likely regulates PYCR2 expression through interactions with RNA-binding proteins or TFs. Next, we comprehensively analyzed the differential TFs and identified 720, 139 and 36 TFs respectively between the three datasets (tumor vs. normal, III-IV vs. I-II, and dead vs. alive) based on the TCGA-COADREAD dataset (Fig. 5C). Notably, three TFs (DDIT3, NR1D1, and ZNF282) exhibited consistent differential expression across those three independent datasets and demonstrated significant associations with adverse clinical outcomes (Fig. 5C, Figure S2 **A**,** C**,** and D**). More importantly, the expression levels of these three TFs are positively correlated with PYCR2, and ZNF282 had the highest correlation (*R* = 0.437, *P* < 0.001) (Figure S2 **B**). RNA-protein interaction prediction using RPISeq [33] (RF classifier score >0.8) identified a positive binding potential between LINC02878 and NR1D1 (RF = 0.85) as well as ZNF282 (RF = 0.80) (Figure S2 **E**). RNA pull-down assays demonstrated direct physical interaction between LINC02878 and ZNF282 in CRC cells, while no binding was observed with other candidate TFs (Fig. 5D). This specific association was further confirmed by RNA immunoprecipitation (RIP) assays (Fig. 5E). Notably, confocal microscopy analysis revealed that LINC02878 overexpression significantly enhanced nuclear co-localization of ZNF282 in SW480 cells, suggesting LINC02878 facilitated ZNF282 nuclear retention or recruitment (Fig. 5F). In addition, ZNF282 protein is mainly located in the cell nucleus based on the Human Protein Atlas (HPA) database [34] (Fig. 5G), which is consistent with our results(Fig. 5F), suggesting that ZNF282 likely exerts its oncogenic regulatory function within the nuclear. We next examined whether ZNF282 transcriptionally regulates PYCR2 expression in CRC cells. As shown in Fig. 5H, our results indicated that the promoter activity of PYCR2 is modulated by varying levels of ZNF282 in CRC cells. Furthermore, we identified three ZNF282 binding sites in the PYCR2 promoter region based on the JASPAR database [21]. Dual-luciferase reporter assays demonstrated that the binding site E2 (−518/−504, CATCCCCGCAAATCG) is essential for ZNF282-mediated transcriptional regulation of PYCR2 promoter activity (Fig. 5J). Consistently, ChIP-qPCR assays indicated the enrichment of ZNF282 in the PYCR2 promoter region (Fig. 5K). Additionally, RT-qPCR and Western blot analyses consistently demonstrated that overexpression or knockdown of ZNF282 significantly increased or inhibited the expression level of PYCR2 in CRC cells, respectively (Fig. 5L-M).

### LINC02878/ZNF282/PYCR2 axis promotes proline synthesis and CRC progression

**Fig. 6:**
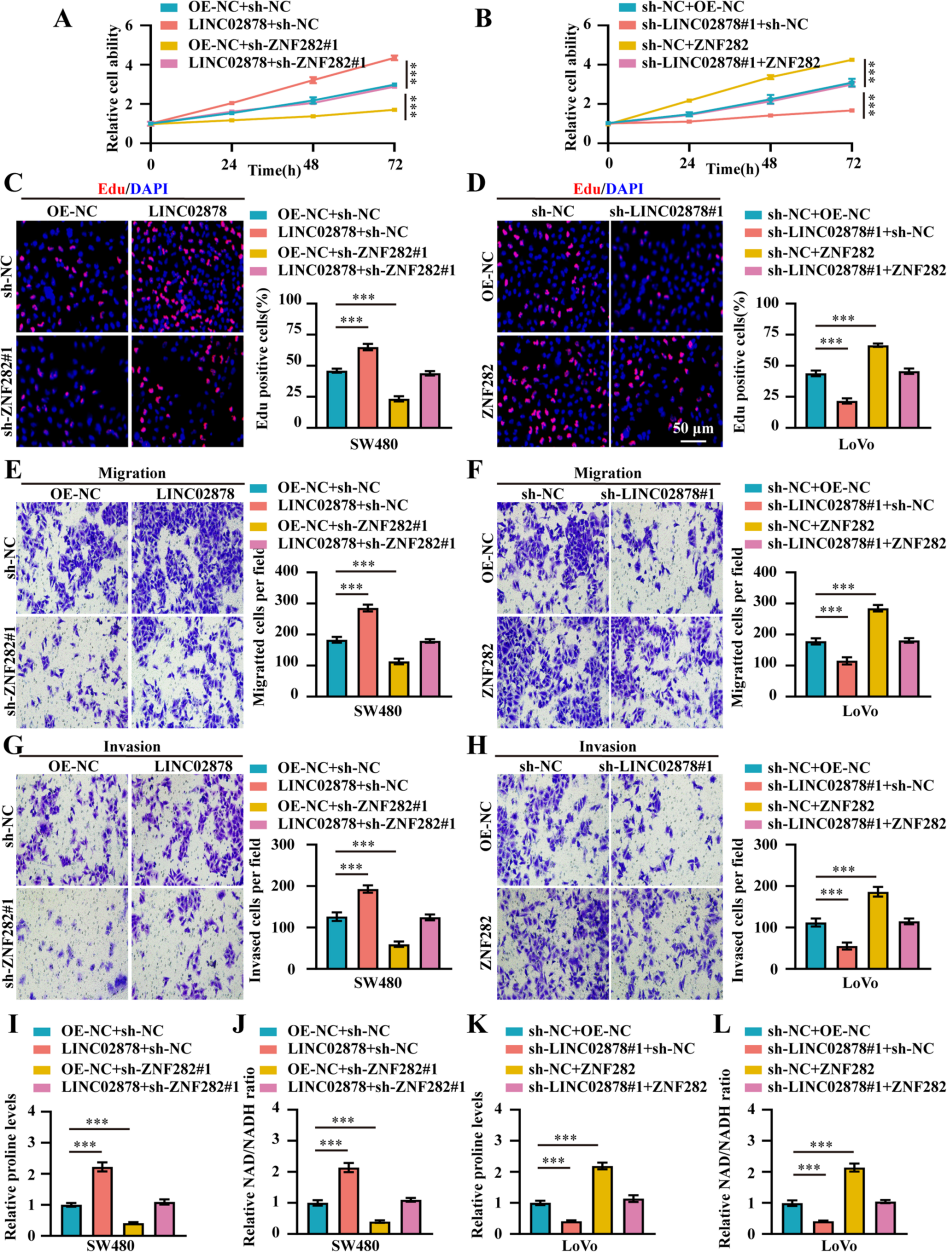
LINC02878/ZNF282/PYCR2 axis promotes proline synthesis and tumor progression in CRC. **(A-B)** CCK-8 detecting the cell viability of SW480 and LoVo cells transfected as indicated, respectively (*n* = 3). (**C-D**) EdU assay evaluating the cell proliferation ability of SW480 and LoVo cells transfected as indicated, respectively (*n* = 3). (**E-H**) Transwell migration and invasion assay detecting the cell migration and invasion ability of SW480 and LoVo cells transfected as indicated, respectively (*n* = 3). (**I-L**) The proline levels, and NAD/NADH ratio in SW480 and LoVo cells stably transfected as indicated, respectively (*n* = 3). *P < 0.05, **P < 0.01, ***P < 0.001

Subsequently, we examined the regulatory mechanisms of the LINC02878/ZNF282/PYCR2 axis in CRC cells. Our results indicated that knockdown of ZNF282 abolished the increases in cell viability, growth, migration, and invasion capacity induced by LINC02878 overexpression in SW480 cells (Fig. 6A, C, E, **and G**). Conversely, overexpression of ZNF282 restored the decreases in cell viability, growth, migration, and invasion capacity caused by stable silencing of LINC02878 in LoVo cells (Fig. 6B, D, F, **and H**). Consistently, knockdown of ZNF282 abolished the increases in proline and NAD/NADH ratio induced by LINC02878 overexpression in SW480 cells (Fig. 6I-J). In addition, overexpression of ZNF282 rescued the inhibitory effect of proline and NAD/NADH ratio induced by LINC02878 knockdown in LoVo cells (Fig. 6K-L).

### Therapeutic knockdown of LINC02878 inhibits CRC progression

**Fig. 7:**
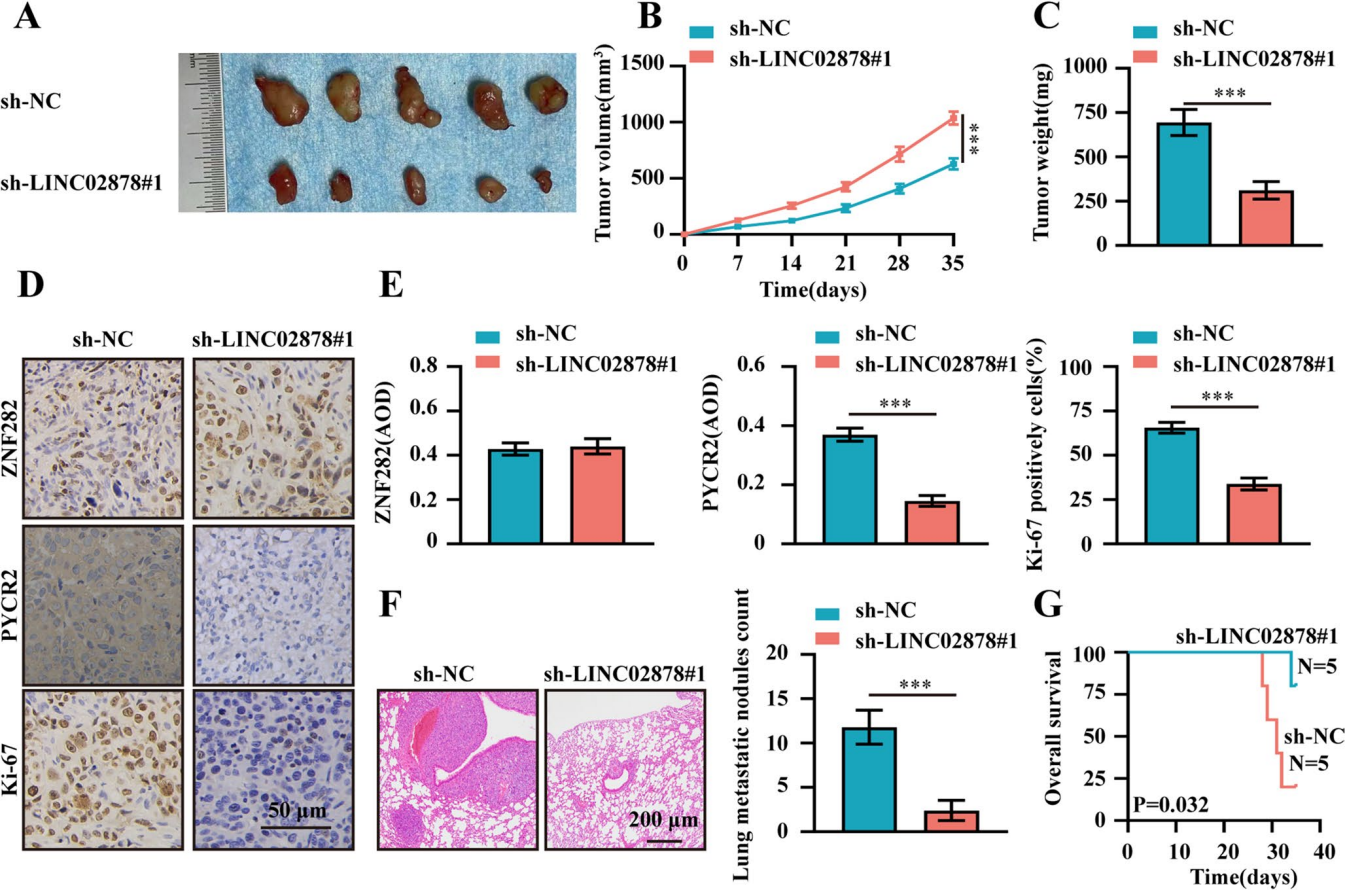
Therapeutic knockdown of LINC02878 inhibits the malignant progression of CRC ***in vivo.*** (**A**) The representative image of xenograft tumors injected LoVo cells stably transfected with sh-NC and sh-LINC02878#1 (*n* = 5 per group). (**B**) Growth curve of xenograft tumors (*n* = 5 per group). (**C**) Tumor weight of xenograft tumors (*n* = 5 per group). (**D-E**) Immunohistochemical analysis was performed to quantify the percentages of ZNF282-, PYCR2-, and Ki-67-positive cells in xenograft tumor tissues (*n* = 5 per group). **(F-G**) Representative H&E-stained lung sections (**F**, left panel, *n* = 5 per group), quantitative analysis of pulmonary metastases (**F**, right panel, *n* = 5 per group), and (**G**) Kaplan-Meier survival curves of nude mice injected with LoVo cells stably expressing sh-NC (control) or sh-LINC02878#1 (*n* = 5 per group). *P < 0.05, **P < 0.01, ***P < 0.001

To assess the therapeutic potential of LINC02878 inhibition in vivo, we established xenograft models by administering stable LINC02878-knockdown LoVo cells via subcutaneous or intravenous injection into nude mice. Compared to controls, LINC02878 silencing significantly attenuated tumor growth rate, reduced tumor weight, and decreased the Ki-67 proliferation index (Fig. 7A). In addition, IHC results confirmed successful downregulation of both LINC02878 and its downstream effector PYCR2 in excised tumors (Fig. 7E). In metastatic models, tail vein injection of LINC02878-depleted cells resulted in significantly fewer pulmonary metastatic nodules and prolonged overall survival (Fig. 7F).

### High expression levels of LINC02878, ZNF282, and the target gene PYCR2 are associated with poor outcomes in CRC

**Fig. 8:**
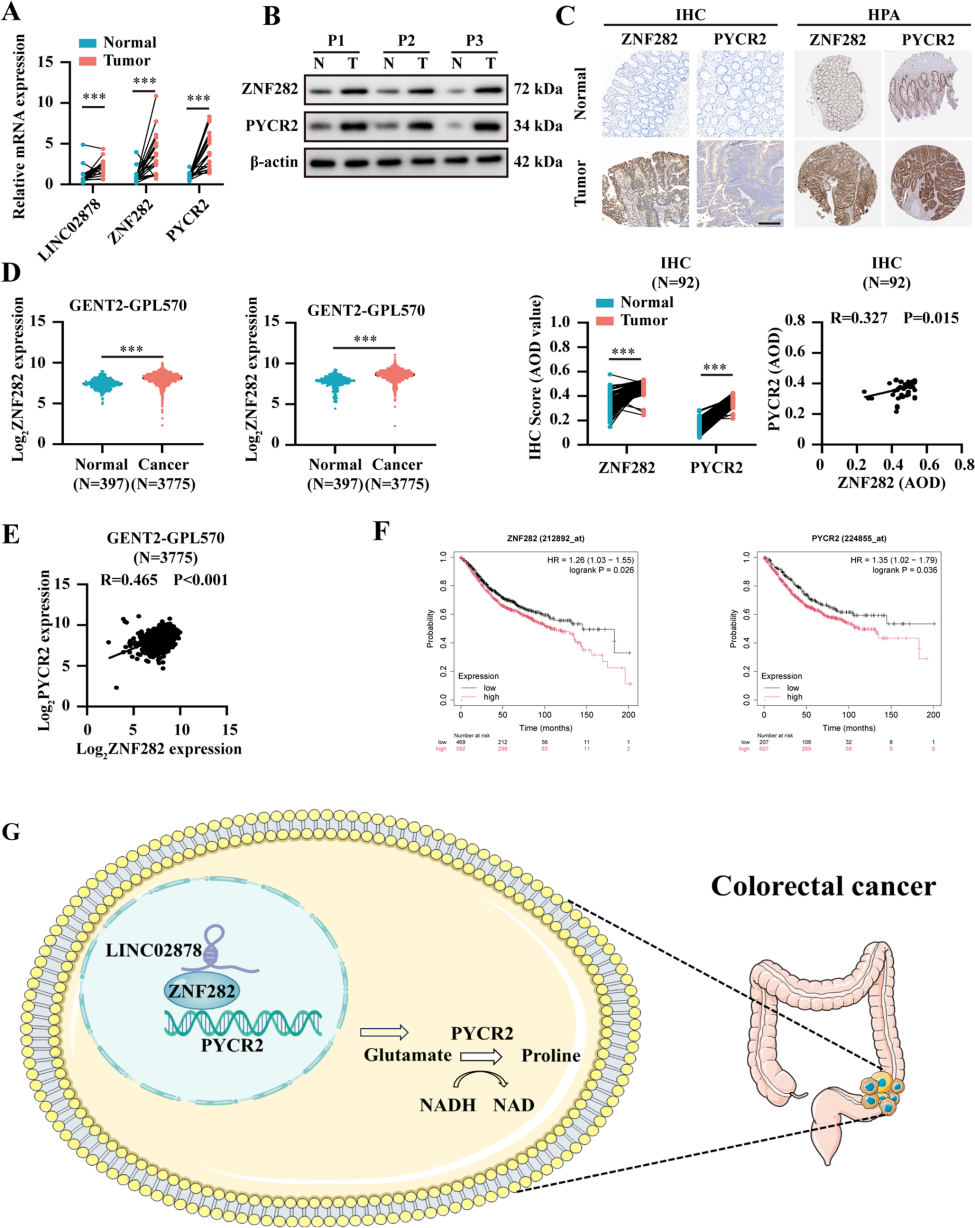
High expression levels of LINC02878, ZNF282, and the target gene PYCR2 are associated with poor outcomes in CRC.** (A) **RT-qPCR (**L, n=50**) and western blot (**M, n=20**) evaluating the mRNA and protein levels between CRC tissues and adjacent normal tissues, respectively. (**C**) Representative IHC images of ZNF282 and PYCR2 based on the IHC (n=92) and HPA database. (**D-E**) The expression levels (**D**) and correlation (**E**) of ZNF282 and PYCR2 in CRC were investigated based on the GENT2 database. (**F**) The survival curves of ZNF282 and PYCR2 in CRC patients based on the Kaplan-Meier Plotter database (https://kmplot.com/analysis/). (**G**) Mechanism schematic diagram: LINC02878 interacts with ZNF282 to transcriptionally activate PYCR2, promoting proline synthesis and tumor progression in colorectal cancer. *P<0.05, **P<0.01,***P<0.001

Subsequently, we assessed the expression profiles of LINC02878, ZNF282, and PYCR2 in CRC specimens. Our results demonstrated that the mRNA levels of LINC02878, ZNF282, and PYCR2 were consistently upregulated in CRC tissues compared with adjacent normal colon tissues (Fig. 8A). Furthermore, Western blot and IHC analyses confirmed significantly elevated protein expression levels of ZNF282 and PYCR2 in CRC tissues (Fig. 8B-C). Critically, independent verification using the HPA database [34] and GENT2 database [35] confirmed our experimental findings of ZNF282 and PYCR2 overexpression in CRC (Fig. 8C). Notably, both IHC results (*N* = 92) and Gene Expression database of Normal and Tumor tissues 2 (GENT2) database (*N* = 3775) [35] analysis demonstrated a positive correlation in the expression patterns of ZNF282 and PYCR2 in CRC specimens (Fig. 8C and E). Notably, elevated expression of ZNF282 and PYCR2 were associated with significantly worse overall survival in CRC patients based on the Kaplan-Meier Plotter database (https://kmplot.com/analysis/) (Fig. 8F). Collectively, our findings demonstrated that elevated expression of LINC02878, ZNF282, and PYCR2 are consistently associated with CRC progression and significantly correlates with adverse clinical outcomes. Importantly, we identify the LINC02878/ZNF282/PYCR2 axis as a critical regulator of proline metabolism in CRC, unveiling its potential as a novel therapeutic target for precision intervention (Fig. 8G).

##  Discussion

CRC remains one of the most prevalent and lethal malignancies worldwide, with increasing incidence in younger populations [36, 37]. Despite advancements in early detection and targeted therapies, metastatic CRC (mCRC) continues to have a poor prognosis due to therapy resistance and tumor heterogeneity [2]. Conventional treatments, including chemotherapy and biologics, often face limitations due to adaptive metabolic reprogramming in cancer cells [38, 39]. Thus, identifying novel molecular drivers provide critical insights into CRC progression and unveils potential therapeutic vulnerabilities.

Recent studies have established that lncRNAs can modulate tumor metabolism by interacting with metabolic enzymes or TFs. For instance, novel lncRNA LOC101928222 synergizes with insulin like growth factor 2 mRNA binding protein 1 (IGF2BP1) to stabilize 3-hydroxy-3-methylglutaryl-CoA synthase 2 (HMGCS2) mRNA through an m6A-dependent pathway, resulting in enhanced cholesterol synthesis and, ultimately, the promotion of CRC development [40]. Another research indicated that LncRNA-HMG binds to p53 and induces the upregulation of solute carrier family 7 member 11 (SLC7A11) and vitamin K epoxide reductase complex subunit 1L1 (VKORC1L1) expression, thereby inhibiting ferroptosis in CRC and promoting chemotherapy resistance [41]. ALMS1 intronic transcript 1 (ALMS1-IT1) inhibits ferroptosis and promotes the growth and metastasis of CRC by activating signal transducer and activator of transcription 3 (STAT3) phosphorylation [42]. Although lncRNAs play a significant regulatory role in CRC, many still require detailed characterization. In this study, we identified LINC02878 as a novel oncogenic lncRNA in CRC and delineated its mechanistic role in metabolic reprogramming. We first observed that LINC02878 is markedly upregulated in CRC tissues and cell lines, with high expression correlating with advanced stage and poor patient survival. Importantly, multivariate analysis established LINC02878 as an independent prognostic factor. These findings suggest that LINC02878 may serve as a valuable diagnostic and prognostic biomarker, consistent with accumulating reports on the prognostic relevance of lncRNAs in CRC malignancies [43]. Gain- and loss-of-function assays revealed that LINC02878 promotes cell viability, proliferation, migration, and invasion. These data support the role of LINC02878 as a bona fide oncogenic driver in CRC. Similar tumor-promoting roles have been reported for other lncRNAs, reinforcing the emerging concept that lncRNAs are integral regulators of CRC progression [43]. Through integrated transcriptomic and clinical analyses, we identified PYCR2 as consistent downstream targets and most relevant effector of LINC02878, indicating that LINC02878 may regulate proline metabolism in CRC through PYCR2, which is consistent with previous reports highlighting the role of PYCR2 as a critical metabolic enzyme in cancer progression [44]. Proline, a non-essential amino acid, plays a pivotal role in collagen synthesis, redox homeostasis, and tumor microenvironment remodeling [15]. Recent studies highlight that PYCR2, a key enzyme in proline biosynthesis, is frequently overexpressed in cancers, and closely related to the malignant progression and poor prognosis of various cancers, including CRC [44–46]. For example, c-Myc transcription regulates PYCR2, thereby activating the AKT signaling pathway to promote the invasion and metastasis of breast cancer [46]. The alkB homolog 5, RNA demethylase (ALKBH5)-PYCR2 positive feedback loop promotes the malignant progression of GBM through proline synthesis [44]. Our findings align with emerging evidence that proline metabolism is hijacked by CRC cancer cells to sustain proliferation, invasion, and metastasis. Although previous studies have demonstrated that PYCR2 is significantly upregulated in CRC and strongly associated with peritoneal invasion and metastatic progression [47], the precise molecular mechanisms underlying PYCR2 overexpression in CRC remain poorly understood. Our findings reveal that LINC02878-mediated transcriptional activation of PYCR2 through its interaction with ZNF282 represents a crucial molecular mechanism underlying PYCR2 overexpression in CRC.

ZNF282, also known as HUB1, was originally identified as a HTLV-I (human T-cell leukemia virus type I) U5RE (U5 repressive element) binding protein [48]. Previous studies have demonstrated the oncogenic role of ZNF282 in various malignancies [13, 14]. Notably, ZNF282 interacts with estrogen receptor α (ERα) and functions as an ERα co-activator, promoting tumor progression in breast cancer [14]. Another study has shown that ZNF282 is a co-activator of E2F1 in esophageal squamous cell carcinoma and promotes tumor progression [13]. Currently, research on ZNF282’s role in tumorigenesis remains limited. Unfortunately, the carcinogenic function and mechanism of ZNF282 in CRC remains unclear. Our study reveals that ZNF282 is significantly upregulated in CRC and associated with poor clinical outcomes. Mechanistically, ZNF282 facilitates proline biosynthesis and promotes CRC progression by transcriptionally activating PYCR2. More importantly, our results suggested the interaction between ZNF282 and PYCR2 provides a mechanistic link between transcriptional regulation and metabolic reprogramming, expanding our understanding of CRC pathogenesis. In additional, our RNA pull-down and RIP assays verified a direct interaction between LINC02878 and ZNF282, suggesting that LINC02878 may regulate PYCR2 transcription through this interaction. Such lncRNA–TF regulatory mechanisms have been widely reported in cancer biology [29]. Taken together, our results identify the LINC02878/ZNF282/PYCR2 axis as a key regulatory cascade driving metabolic reprogramming in CRC. Targeting this axis may therefore represent a novel therapeutic strategy, complementing existing approaches against metabolic vulnerabilities in colorectal cancer [49]. Furthermore, in vivo silencing of LINC02878 significantly impaired tumor growth and metastasis, supporting its therapeutic potential. These findings further emphasize the clinical relevance of targeting lncRNA-mediated metabolic pathways in CRC treatment.

Despite these advances, several limitations should be acknowledged. The prognostic role of LINC02878 requires validation in larger, independent patient cohorts. Additional regulators beyond ZNF282 may also contribute to PYCR2 activation. Furthermore, the clinical translation of LINC02878-targeted therapies will require optimization of delivery systems and rigorous safety evaluation. Future work should address these issues to fully assess the therapeutic potential of targeting the LINC02878 axis.

In summary, our study establishes LINC02878 as a clinically significant oncogenic lncRNA in CRC, with its overexpression strongly correlating with adverse patient outcomes. Mechanistically, we demonstrate that LINC02878 physically interacts with the ZNF282 to form a regulatory axis that transcriptionally activates PYCR2. This LINC02878/ZNF282/PYCR2 signaling cascade drives proline anabolism, thereby fueling tumor metabolic reprogramming and fostering aggressive malignant progression in CRC. These findings not only elucidate a novel lncRNA-mediated metabolic mechanism in CRC pathogenesis but also highlight the therapeutic potential of targeting the LINC02878-ZNF282-PYCR2 axis. Given the critical role of proline metabolism in cancer cell survival and metastasis, our work provides a conceptual framework for developing precision therapies against proline-dependent CRCs with LINC02878 overexpression.

## Supplementary Information

Below is the link to the electronic supplementary material.

Supplementary Material 1 (DOCX 15.7 KB)

Supplementary Material 2 (DOCX 647 KB)

Supplementary Material 3 (DOCX 1.03 KB)

## Data Availability

The original data are included in the article. For additional information, inquiries can be directed to the corresponding authors.

## Abbreviations

ALKBH5: AlkB homolog 5, RNA demethylase
ALMS1-IT1: ALMS1 intronic transcript 1
Erα: Estrogen receptor α
HMGCS2: 3-hydroxy-3-methylglutaryl-CoA synthase 2
IGF2BP1: Insulin like growth factor 2 mRNA binding protein 1
mCRC: Metastatic CRC
SIRT3: Sirtuin 3
SLC7A11: Solute carrier family 7 member 11
SP100-AS1: lncRNA targeting antisense sequence of SP100 gene
TAF15: TBP-associated factor 15
TPD52: Tumor protein D52
VKORC1L1: Vitamin K epoxide reductase complex subunit 1L1
ZIC2: Zic family zinc finger 2
ZNF282: Zinc finger protein 282
CCNA1: Cyclin A1
CDC6: Cell division cycle 6
COADREAD: Colorectal adenocarcinoma
CoCoA: Coiled-coil coactivator
CRC: Colorectal cancer
DEGs: Differentially expressed genes
E2F1: E2F transcription factor 1
ESCC: Esophageal squamous cell carcinoma
GO: Gene Ontolog
GSEA: Gene set enrichment analysis
H&E: Hematoxylin& eosin
HK2: Hexokinase 2
HPA: Human Protein Atlas
IHC: Immunohistochemistry
KEGG: Kyoto Encyclopedia of Genes and Genomes
KLF7: KLF transcription factor 7
lncRNAs: Long non-coding RNAs
LOXL1-AS1: LOXL1 antisense RNA 1
MZF1-AS1: MZF1 antisense RNA 1
OS: Overall survival
PARP1: Poly (ADP-ribose) polymerase 1
PFI: Progression-free interval
PYCR1: Pyrroline-5-carboxylate reductase 1
PYCR2: Pyrroline-5-carboxylate reductase 2
RIP: RNA Immunoprecipitation
RNA-FISH: RNA fluorescence in situ hybridization
RNF14: Ring finger protein 14
ROC: Receiver operating characteristic
RT-qPCR: Reverse transcription-quantitative polymerase chain reaction
SNHG4: Small nucleolar RNA host gene 4
SOX2: SRY-box transcription factor 2
STAT3: Transducer and activator of transcription 3
TCGA: The Cancer Genome Atlas
TFs: Transcription factors
ZNF282: Zinc finger protein 282
