# Supplementary material for: LINC02878/ZNF282/PYCR2 axis promotes proline synthesis and tumor progression in colorectal cancer
Source: Cell Mol Life Sci. 2025 Dec 2;83(1):25. doi: 10.1007/s00018-025-05968-3 (PMC12775219; doi:10.1007/s00018-025-05968-3)
Supplement: Supplementary file 1 [file 18_2025_5968_MOESM1_ESM.docx]

Table S1 Primer sets used for RT- qPCR 

| Gene symbol | Sequence (5’-3’) | Product length | PCR efficiency (E, %) | Source |
| --- | --- | --- | --- | --- |
| LINC02878 | Forward: TGCGGCGGATCATAAGGACT | 157 | 98.2 | Oligo 7 |
| Reverse: CATCACCCACGTCTCTGCTG |
| PSMD4 | Forward: CCCAGCAGGATGCTGTCAAC | 169 | 95.6 | Oligo 7 |
| Reverse: ATCTTGCCCTTGGGTTGGAC |
| PYCR2 | Forward: CCCCAGAAATGAACCTGCCC | 157 | 101.3 | Oligo 7 |
| Reverse: ACGTCGGCCCCAATCTCATC |
| SEM1 | Forward: GAGTTCCCTGCCGAAGACTG | 173 | 97.4 | Oligo 7 |
| Reverse: GGAGGTGGGCATCTTCTCAG |
| ZNF282 | Forward: GAAAAGACGGCCGTGGAATT | 131 | 96.8 | Oligo 7 |
| Reverse: AAGTTCCTGTTGCGCAGCAA |
| β-actin | Reverse: GGGCATGGGTCAGAAGGATT | 158 | 99.1 | Oligo 7 |
| Reverse: GAGCCACACGCAGCTCATTG |
