# Supplementary material for: LINC02878/ZNF282/PYCR2 axis promotes proline synthesis and tumor progression in colorectal cancer
Source: Cell Mol Life Sci. 2025 Dec 2;83(1):25. doi: 10.1007/s00018-025-05968-3 (PMC12775219; doi:10.1007/s00018-025-05968-3)
Supplement: Supplementary file 3 [file 18_2025_5968_MOESM3_ESM.docx]

Figure S2 DDIT3, NR1D1, and ZNF282 are highly expressed in CRC and associated with a poor prognosis. (A) The expression of DDIT3, NR1D1, and ZNF282 are significantly increased in tumor tissues (left panel), Ⅲ-Ⅳ stage (middle panel) and death groups (right panel) based on the TCGA-COADREAD. (B) Scatter plot showing the correlation of the expression levels of PYCR2 and DDIT3, NR1D1, and ZNF282 based on the TCGA-COADREAD, respectively. (C-D) The K-M curve suggesting the overall survival (OS) and progression free survival (PFI) of DDIT3, NR1D1, and ZNF282 based on the TCGA-COADREAD, respectively. (E) RNA-protein interaction prediction using RPISeq (RF classifier score > 0.8). *P<0.05, **P<0.01, ***P<0.001.
